# SARS-CoV-2 Vaccination Rate and SARS-CoV-2 Infection of Health Care Workers in Aerosol-Generating Medical Disciplines

**DOI:** 10.3390/jcm11102751

**Published:** 2022-05-12

**Authors:** Anna Muzalyova, Alanna Ebigbo, Maria Kahn, Stephan Zellmer, Albert Beyer, Jonas Rosendahl, Johannes Zenk, Bilal Al-Nawas, Roland Frankenberger, Juergen Hoffmann, Christoph Arens, Frank Lammert, Claudia Traidl-Hoffmann, Helmut Messmann, Christoph Roemmele

**Affiliations:** 1 Clinic for Internal Medicine III—Gastroenterology and Infectious Diseases, University Hospital of Augsburg, 86156 Augsburg, Germany; alanna.ebigbo@uk-augsburg.de (A.E.); maria.kahn@gmx.de (M.K.); stephan.zellmer@uk-augsburg.de (S.Z.); helmut.messmann@uk-augsburg.de (H.M.); christoph.roemmele@uk-augsburg.de (C.R.); 2 Medical Practice for Gastroenterology and Gastrointestinal Oncology, 84503 Altoetting, Germany; beyer@innere.org; 3 Clinic for Internal Medicine I—Gastroenterology and Pneumology, University Hospital Halle, 06120 Halle (Saale), Germany; jonas.rosendahl@uk-halle.de; 4 Department of Otorhinolaryngology, Head and Neck Surgery, University Hospital Augsburg, 86156 Augsburg, Germany; johannes.zenk@uk-augsburg.de; 5 Clinic and Polyclinic for Oral and Maxillofacial Surgery, Plastic Surgery, University Hospital Mainz, 55131 Mainz, Germany; al-nawas@uni-mainz.de; 6 Department for Operative Dentistry, Endodontics, and Pediatric Dentistry, Philipps University Marburg and University Hospital Giessen and Marburg, 35039 Marburg, Germany; frankbg@med.uni-marburg.de; 7 Department of Oral and Maxillofacial Surgery, University Hospital Heidelberg, 69120 Heidelberg, Germany; juergen.hoffmann@med.uni-heidelberg.de; 8 Department of Otorhinolaryngology, Head and Neck Surgery, University Hospital Magdeburg, 39120 Magdeburg, Germany; christoph.arens@med.ovgu.de; 9 Saarland University Medical Center, Department of Medicine II, Saarland University, 66421 Homburg, Germany; frank.lammert@uks.eu; 10 Hannover Medical School (MHH), 30625 Hannover, Germany; 11 Department of Environmental Medicine, Faculty of Medicine, University of Augsburg, 86156 Augsburg, Germany; claudia.traidl-hoffmann@uk-augsburg.de

**Keywords:** aerosol-generating procedures, SARS-CoV-2, perceived risk of infection, vaccination rate

## Abstract

Healthcare workers (HCW) who perform aerosol-generating procedures (AGP) are at high risk of SARS-CoV-2 infection. Data on infection rates and vaccination are limited. A nationwide, cross-sectional study focusing on AGP-related specialties was conducted between 3 May 2021 and 14 June 2021. Vaccination rates among HCW, perception of infection risk, and infection rates were analyzed, focusing on the comparison of gastrointestinal endoscopy (GIE) and other AGP-related specialties (NON-GIE), from the beginning of the pandemic until the time point of the study. Infections rates among HCW developed similarly to the general population during the course of the pandemic, however, with significantly higher infections rates among the GIE specialty. The perceived risk of infection was distributed similarly among HCW in GIE and NON-GIE (91.7%, CI: 88.6–94.4 vs. 85.8%, CI: 82.4–89.0; *p* < 0.01) with strongest perceived threats posed by AGPs (90.8%) and close patient contact (70.1%). The very high vaccination rate (100–80%) among physicians was reported at 83.5%, being significantly more frequently reported than among nurses (56.4%, *p* < 0.01). GIE had more often stated very high vaccination rate compared with NON-GIE (76.1% vs. 65.3%, *p* < 0.01). A significantly higher rate of GIE was reported to have fewer concerns regarding infection risk after vaccination than NON-GIE (92.0% vs. 80.3%, *p* < 0.01).

## 1. Introduction

Novel severe acute respiratory syndrome of corona virus type 2 (SARS-CoV-2) was first reported in Wuhan, China, in late 2019 and spread rapidly, resulting in a global COVID-19 pandemic officially declared by the World Health Organization on 11 March 2020 [1]. The ongoing pandemic has caused over 490 million cases of COVID-19 and has claimed more than 6 million lives worldwide (as of 6 April 2022) [2]. Since the beginning of the COVID-19 pandemic, Germany has experienced a wavelike development of infection and mortality with several peaks.

Initially, herd immunity of the general population was set as a target to mitigate the spread of SARS-CoV-2 [3]. Effective and safe vaccines can make a decisive contribution in defeating the pandemic. The European Commission has granted Europe-wide approval to the COVID-19 vaccine developed by BioNTech/Pfizer on 21 December 2020, giving the go-ahead to the start of a vaccination campaign in Germany on 27 December 2020 [4]. Due to the limited availability of the vaccine, a prioritization of different groups of people was made based on the risk associated with their health condition and potential exposure to the virus [3,4]. As healthcare workers (HCW) are exposed to a high risk of infection due to direct patient contact [5] or certain high-risk examinations, such as aerosol-generating procedures [5,6], they were assigned to a high priority in the vaccination campaign in Germany [7]. However, vaccination alone cannot be considered as a panacea in defeating the pandemic due to the insufficient vaccination rates and the emergence and spread of variants of concern (VOC), increasing transmission and severity of the disease [8,9]. Consequently, a holistic approach in defeating the SARS-CoV-2 pandemic is required.

The present study is a continuation of the CoREM-NUM study being part of a collaborative B-FAST project of the Network University Medicine (NUM) initiated by the German Federal Ministry of Education and Research (BMBF). In the course of the study, the University Hospital Augsburg collected data on AGP-associated medical specialties such as gastrointestinal endoscopy, otorhinolaryngology, oral and maxillofacial surgery, and dental medicine.

The primary objective of this study was to investigate the vaccination and infection rates among HCW in the course of the COVID-19 pandemic.

## 2. Materials and Methods

### 2.1. Study Design

The present study is a descriptive, nationwide cross-sectional study conducted in Germany. As it is a continuation of the CoREM-NUM study, the objective focuses on risk assessment of aerosol-generating procedures in the context of the COVID-19 pandemic. Details and results on the first part of the CoREM-NUM study were reported in previous publications [10,11,12,13].

Furthermore, the research interest was focused on the four medical disciplines: gastrointestinal endoscopy (GIE), otolaryngology (ORL), oral and maxillofacial surgery (OMS), dentistry (DM), and comparison of the GIE specialty with other AGP-related medical disciplines. Therefore, data on ORL, OMS and DM are presented aggregated and denoted as NON-GIE. The study questionnaire was designed based on expert advice as well as insight from the previous studies, and implemented in UniPark© (Material S1). The questionnaire particularly sought information on the prevalence of SARS-CoV-2 infection from the beginning of the pandemic (second calendar quarter 2020) until the time point of the study (second calendar quarter 2021), vaccination rates, HCWs’ perceived risk of infection due to professional activity and its probable change due to vaccination. Vaccination rates of HCW were inquired on a five-step scale comprising the following categories: very high (100–80%), high (80–60%), average (60–40%), low (40–20%), and very low (20–0%) rates.

Study participants were defined as medical facilities comprising hospitals or private practices belonging to one of the four pre-defined medical disciplines of research interest. Therefore, study participants not being clearly attributed to one of the four medical specialties were considered ineligible and excluded from further data analysis. Potential study participants were contacted via e-mail distribution of the respective professional societies such as the German Gastroenterology Association (DGVS), the German Society of Dentistry and Oral Medicine (DGZMK), the German Society of Oral and Maxillofacial Surgery (DGMKG), the German Society of Otorhinolaryngology, Head and Neck Surgery (DGHNO-KHC), and the Professional Association of Gastroenterologists in Private Practice (bng) and invited to fill in an online questionnaire. Participation in the online survey was possible between 3 May and 14 June 2021. The study site had no direct contact to the potential study site assuring anonymity of the study participants.

### 2.2. Statistical Analysis

The data management and statistical analysis were conducted using SPSS^©^ version 27.0. The categorical variables such as medical specialty, healthcare delivery setting, estimated vaccination rates and expectations regarding vaccination are presented as absolute frequencies and percentages. HCW infection rates were calculated as the proportion of the absolute number of SARS-CoV-2 positive infections among HCW as reported by the respective category of medical facilities to the underlying total of the study population in the same category and expressed as percentage. The infection rates in the German population were calculated using data from the Robert Koch Institute (RKI) [14], the leading governmental institution for infection surveillance in Germany, by summing up absolute number of infections per quarter in the general population and setting it in relation to the number of inhabitants in Germany (83.1 million) [15]. The relationships between nominal-scaled variables were tested inferentially using Chi-square independence tests or Fisher’s exact test.

## 3. Results

### 3.1. Sample Characteristics

In total, 849 medical facilities participated with over 10,000 HCW in the second part of the CoREM-NUM survey, of which 832 (98.0%) were clearly attributed to one of the four pre-defined medical specialties. Most of the study participants were from private practices 76.6% (*n* = 637), whereas hospitals constituted 23.4% (*n* = 195) of the total study population (Table 1). Among private practices the largest proportion was represented by NON-GIE specialties (52.4%, *n* = 436). Among participating hospitals GIE was predominant with 17.5% (*n* = 146) of facilities belonging to this medical discipline.

### 3.2. HCW Infection Rate

**Figure 1:**
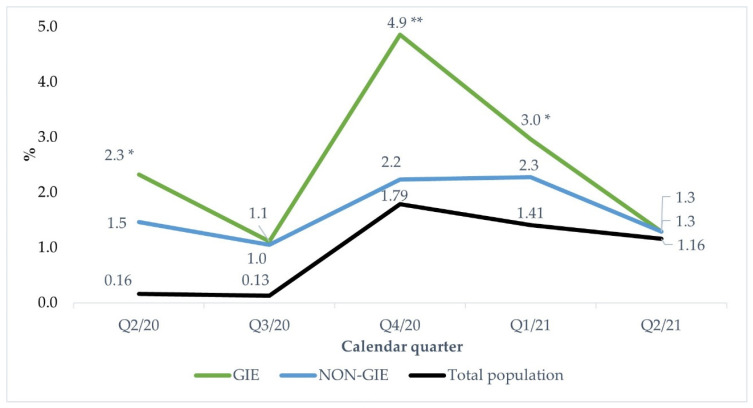
SARS-CoV-2 infection rates among HCW by medical specialty from 2th Quarter 2020 to 2th Quarter 2021. GIE: gastrointestinal endoscopy; NON-GIE: other aggregated aerosol-generating specialties such as otolaryngology, oral- and maxillofacial surgery and dental medicine. ** Significance level *p* < 0.01; * Significance level *p* < 0.05—comparison of GIE vs. NON-GIE specialties.

The second and third quarters of 2020 were characterized by a stable low infection rate in the general population accounting for 0.16% and 0.13%, respectively (Figure 1). In the fourth quarter, 2020 infection rates increased up to 1.79%, highlighting the peak of infection rates followed by a steady decreasing trend in the first half of 2021.

The HCW infection rates in the GIE specialty followed the pattern of the infection rates outside the medical facilities with the peak of infection rates occurring in the fourth quarter 2020 and experiencing a sharp decline in the first half of 2021. However, compared with the general population, the peak of infections in GIE was proportionally higher. The GIE specialty was more strongly affected by the COVID-19 pandemic, as it experienced significantly higher infection rates in the second (2.3% vs. 1.5%, *p* < 0.01) and fourth quarter 2020 (4.9% vs. 2.2%, *p* < 0.01), as well as in the first quarter 2021 compared with NON-GIE specialties (3.0% vs. 2.3%, *p* < 0.05). On the contrary, the infection rates of NON-GIE specialty were more similar to infection rates in the general population, although the decrease of infection rates was delayed by one calendar quarter in comparison to GIE or the general population.

### 3.3. Perceived Risk of Infection among HCW

**Figure 2:**
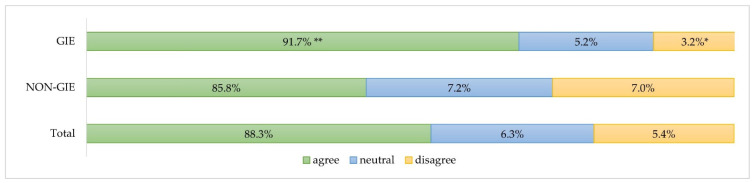
Assessment of the statement: “HCW are at higher risk of SARS-CoV-2 infection compared with the general population” by medical specialty. GIE: gastrointestinal endoscopy; NON-GIE: other aggregated aerosol-generating specialties such as otolaryngology, oral- and maxillofacial surgery and dental medicine. ** Significance level *p* < 0.01; * Significance level *p* < 0.05.

Eighty-eight percent of study participants (*n* = 737) stated they were exposed to a higher risk of SARS-CoV-2 infection than the general population. Accordingly, 91.7% (*n* = 320) of the GIE study participants have stated they perceived a higher risk of infection due to their professional activity, whereas agreement among NON-GIE in this regard was made by a significantly smaller proportion of the participants (85.8%, *n* = 417; *p* < 0.01) (Figure 2).

The most anticipated risk factor for work-related SARS-CoV2 infection were AGPs with 92.7% (*n* = 771), followed by contact frequency determined by the professional activity (74.9%, *n* = 623) and compelled close contact to the patients (71.5%, *n* = 595) (Table 2). The least concerns were caused by the sensitivity of the rapid antigen test 15.7% (*n* = 131) or unreasonable patients 32.8% (*n* = 273).

### 3.4. Vaccination Rates among HCW

**Figure 3:**
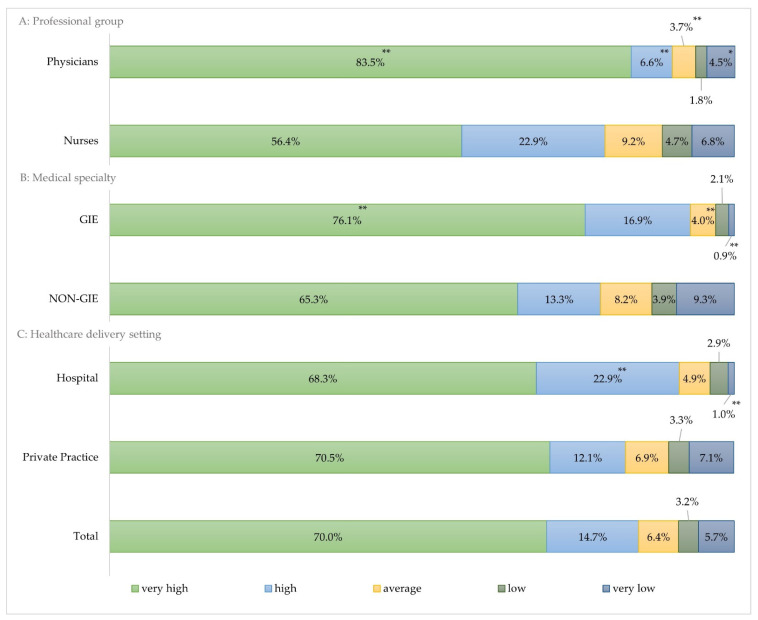
Vaccination rate reported by study participants: (**A**) vaccination rate by professional group; (**B**) Vaccination rate by medical specialty; (**C**) Vaccination rate by health care delivery setting. GIE: gastrointestinal endoscopy; other aggregated aerosol-generating specialties such as otolaryngology, oral- and maxillofacial surgery and dental medicine. ** Significance level *p* < 0.01; * Significance level *p* < 0.05.

Overall, 70.0% (*n* = 1188) of study participants stated the vaccination rate of HCW to be very high (100–80%), followed by 14.7% (*n* = 250) with a high vaccination level (80–60%). The proportion of those with a very low vaccination level (20–0%) was only 5.7% (*n* = 96) (Figure 3).

In general, physicians have a higher vaccination rate than nurses in Germany. Therefore, 83.5% (*n* = 709) of participating facilities stated the vaccination rate of physicians was very high (80–100%), whereas a significantly smaller percentage of study participants (56.4%, *n* = 479; *p* < 0.01) stated they had the same vaccination rate for nurses (Figure 3).

Regarding differences specific for medical disciplines, 76.1% (*n* = 531) of GIE assessed the vaccination rate of their HCW to be very high (100–80%). In contrast, a significantly smaller proportion of the NON-GIE study participants (65.3%, *n* = 317; *p* < 0.01) chose the same category for the vaccination rate of their workforce. Furthermore, the largest group of HCWs having a very low vaccination rate (20–0%) was observed in the NON-GIE specialty, accounting for 9.3% (*n* = 45) %), which is significantly larger as compare to the GIE (*p* < 0.01) (Figure 3).

Overall, 68.3% (*n* = 280) of hospitals estimated the vaccination rate among HCW to be very high (100–80%), whereas a slightly larger proportion of 70.5% (*n* = 905) of private practices has chosen the same category (*p* = 0.424). A high vaccination rate (80–60%) was reported by a significantly larger proportion of hospitals as compared to private practices (22.9%, *n* = 94 vs. 12.1%, *n* = 156, *p* < 0.01).

### 3.5. Vaccination Rates Depending on Perceived Risk of SARS-CoV-2 Infection

**Figure 4:**
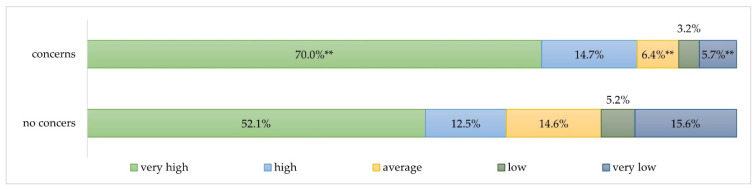
Vaccination rate depending on concerns regarding perceived risk of SARS-CoV-2 infection compared with the general population. ** Significance level *p* < 0.01; * Significance level *p* < 0.05.

Overall, study participants having concerns regarding being at a higher risk of SARS-CoV-2 infection due to their professional activity had higher HCW vaccination rates. Therefore, 70.0% (*n* = 1188) of study participants seeing themselves at a higher risk of infection have reported a very high vaccination rate in their departments, whereas those, having no concerns in this regard chose the same vaccination rate significantly less often with 52.1% (*n* = 50, *p* < 0.01) (Figure 4).

A similar tendency was observed when comparing professional groups (Material S2). In medical departments with higher perceived infection risk both physicians and nurses were better vaccinated than in medical department with no perception of increased infection risk.

### 3.6. Perceived Vaccination Protection among Fully Vaccinated Study Participants

Overall, 85.3% (*n* = 669) of study participants stated to have less concerns due to vaccination (Table 3). However, HCW in the NON-GIE specialties were significantly more skeptical regarding the effectivity of the vaccine. Accordingly, 19.7% (*n* = 88) of NON-GIE and only 8.0% (*n* = 27) of GIE study participants were seen to have the same concerns of getting infected despite being fully vaccinated (*p* < 0.01).

## 4. Discussion

Vaccination of the population is considered to be the most important pillar in fighting the COVID-19 pandemic. Indeed, a recent publication evaluating the vaccination campaign in Germany based on clinical COVID-19 cases from beginning of the vaccination campaign from 27 December 2020 until 14 July 2021 revealed that vaccination prevented a substantial number of severe and lethal COVID-19 cases [4]. According to our results, 70.0% of questioned medical facilities stated the vaccination rate of their physicians was in the range of 80–100%.

### 4.1. Vaccination Rates in Professional Groups

The vaccination rate found in the present study demonstrated a higher acceptance rate of SARS-CoV-2 vaccination among physicians compared with the general population in Germany. Accordingly, the estimated acceptance in the general population was around 70.0% at the beginning of the pandemic [16,17]. However, a substantially higher vaccination readiness was found among physicians with more than 80.0% of study participants stating very high vaccination rates (100–80%) in this professional group. In contrast, nurses lag behind with very high vaccination rates reported by only 55.0% of medical facilities. In line with this, a study of Dror et al. (2020) revealed a higher vaccine acceptance among physicians compared with nurses, with 78.1% to 61.1%, respectively [18]. A similar result was found in a French survey with nurses being less keen to accept vaccination against SARS-CoV-2 than physicians [19]. Still little is known about the extent and nature of the SARS-CoV2 vaccination hesitancy, especially among HCW. Nevertheless, a recent publication showed that a higher level of education or the perceived risk of becoming infected are factors that increase vaccination uptake [20]. Evidence on the risk of infection depending on the professional group within a medical facility is inconsistent [21]. Some studies suggest nursing-related occupations to be associated with a higher risk of SARS-CoV-2 infection compared with physicians [22]. However, as direct contact with patients being a risk factor [23], and especially in consideration of high number of asymptotic SARS-CoV-2 courses [23,24], the vaccination rate among nurses appears to be insufficient. This implies a need for targeted awareness campaigns that focuses specifically on this professional group. In order to counter low vaccination rates, the German government has issued compulsory vaccination in the healthcare sector on 15 March 2022.

As HCW are at the frontline of the pandemic, the World Health Organization has identified HCW as a priority group for SARS-CoV-2 vaccination [25]. However, due to limited availability of the vaccine, the German Standing Committee on Vaccination (STIKO) has assigned medical specialties to different priority groups based on their presumed risk of exposure to SARS-CoV-2 infection [3]. According to our study, the GIE specialty has shown a higher vaccination rate than NON-GIE specialties. GIE HCW received first priority in the vaccination campaign getting access to the vaccine starting from 27 December 2020. Since medical disciplines belonging to NON-GIE such as OMS/DM and ORL were identified as second priority, their vaccination started at the end of the first quarter of 2021. Therefore, the lower HCW vaccination rates found in this manuscript might be attributed to later vaccination start and might catch up during the ongoing vaccination campaign.

Higher perceived risk of getting infected with SARS-CoV-2 is another facilitating factor of SARS-CoV-2 vaccination [20], which was assessed as slightly higher by GIE specialties compared with NON-GIE.

### 4.2. Infection Rate

Nevertheless, the infection rates among HCW in the GIE specialty were higher than in NON-GIE throughout the whole study period. Multiple reasons could be responsible for this observation. Firstly, the higher infection rates might be attributed to GIE departments taking care of COVID-19 patients. Secondly, abdominal symptoms may occur during the clinical course of SARS-CoV-2 disease, requiring an assessment by a GIE specialist [26]. Finally, non-elective procedures are more often performed in hospitals, being in this survey predominantly represented by GIE specialty.

Considering the NON-GIE specialty, the peak of infection rates occurred delayed by one calendar quarter compared with GIE, followed by a declining trend occurring firstly in the second quarter 2021. Typical symptoms of COVID-19 such as fever, cough, headache, rhinitis and sore throat [27] are often associated with usual infectious respiratory diseases. ORL private practices, being a substantial part of the NON-GIE study population, might be the first contact point for patients being unaware of their COVID-19 disease. Therefore, the delayed peak of infection rates in the NON-GIE might be associated with a later start of the vaccination campaign in specialty.

### 4.3. Limitations

Like other cross-sectional studies, our study has some limitations. Due to the recruitment strategy via the professional associations, a selection bias cannot be ruled out. In particular, DM and OML were not represented in large numbers comprising only five facilities. Another shortcoming of the study is that this study was cross-sectional, inquiring information over a considerable period in the past comprising three calendar quarters in 2020 and two calendar quarters of 2021. Moreover, insights presented are based on the assessments and judgments made for a private practice or a hospital ward and its workforce by one individual. Furthermore, the vaccination rates were assessed using a 5-step Likert-scale, comprising a span of 20%, which makes an inference-statistical comparison of infection rates problematic.

Despite the above-mentioned limitations this is the first study to present vaccination rates of HCW in Germany in combination with prevalence of SARS-CoV-2 infection among HCW over the course of the COVID-19 pandemic.

## Figures and Tables

**Table 1:** Absolute number and distribution of facility type and medical specialty.

	Hospital			Private Practice			Total		
	*n*	%	HCW Number	*n*	%	HCW Number	*n*	%	HCW Number
GIE	146	17.5	2303	201	24.2	2184	347	41.7	4487
NON-GIE	49	5.9	2907	436	52.4	3818	485	58.3	6725
Total	195	23.4	5210	637	76.6	6002	832	100.0	11,212

GIE: gastrointestinal endoscopy; NON-GIE: other aggregated aerosol-generating specialties such as otolaryngology, oral- and maxillofacial surgery and dental medicine; *n* number of facilities.

**Table 2:** Main anticipated infection threats of HCW.

	GIE		NON-GIE		Total	
	*n*	%	*n*	%	*n*	%
AGP treatment	321	92.0	450	92.6	771	90.8
Job-related contact frequency	259	74.2	364	74.9	623	73.4
Close contact with patients	244	69.9	351	72.2	595	70.1
Long stay with patients in the treatment room	192	55.0	275	56.6	467	55.0
Frequently asymptomatic courses of COVID-19 infection	179	51.3	277	57.0	456	53.7
Variants of concern (VOC)	105	30.1	173	35.6	278	32.7
Unreasonable patients	118	33.8	155	31.9	273	32.2
Low sensitivity of the rapid antigen test	59	16.9	72	14.8	131	15.4

GIE: gastrointestinal endoscopy; NON-GIE: other aggregated aerosol-generating specialties such as otolaryngology, oral- and maxillofacial surgery and dental medicine.

**Table 3:** Perceived risk of SARS-CoV-2 infection after vaccination by medical specialty.

	GIE		NON-GIE		Total	
	*n*	%	*n*	%	*n*	%
Less concerns	310	92.0 **	359	80.3	669	85.3
Same concerns	27	8.0	88	19.7	115	14.7

GIE: gastrointestinal endoscopy; NON-GIE: other aggregated aerosol-generating specialties such as otolaryngology, oral- and maxillofacial surgery and dental medicine. ** Significance level *p* < 0.01; * Significance level *p* < 0.05.

## Data Availability

Data are available upon reasonable request.

## Supplementary Materials

The following supporting information can be downloaded at: https://www.mdpi.com/article/10.3390/jcm11102751/s1, Material S1. Study questionnaire. Material S2. Vaccination rate depending on perceived risk of SARS-CoV-2 infection.

## References

[1] Cucinotta D., Vanelli M. (2020). WHO Declares COVID-19 a Pandemic. Acta Biomed..

[2] (2021). John Hopkins Coronavirus Resource Center. https://coronavirus.jhu.edu/map.html.

[3] Vygen-Bonnet S., Koch J., Bogdan C., Harder T., Heininger U., Kling K., Littmann M., Meerpohl J., Meyer H., Mertens T. (2020). Beschluss und Wissenschaftliche Begründung der Ständigen Impfkommission (STIKO) für die COVID-19-Impfempfehlung. Epid. Bull..

[4] Waize M., Scholz S., Wichmann O., Harder T., Treskova-Schwarzbach M., Falman A., Weidemann F., Karch A., Lange B., Kuhlmann A. (2021). Die Impfung gegen COVID-19 in Deutschland zeigt eine hohe Wirksamkeit gegen SARS-CoV-2-Infektionen, Krankheitslast und Sterbefälle. Epid. Bull..

[5] Jespersen S., Mikkelsen S., Greve T., Kaspersen K.A., Tolstrup M., Boldsen J.K., Redder J.D., Nielsen K., Abildgaard A.M., Kolstad H.A. (2020). SARS-CoV-2 seroprevalence survey among 17,971 healthcare and administrative personnel at hospitals, pre-hospital services, and specialist practitioners in the Central Denmark Region. Clin. Infect. Dis..

[6] Chou R., Dana T., Buckley D.I., Selph S., Fu R., Totten A.M. (2020). Update Alert 3: Epidemiology of and Risk Factors for Coronavirus Infection in Health Care Workers. Ann. Intern. Med..

[7] Sallam M. (2021). COVID-19 Vaccine Hesitancy Worldwide: A Concise Systematic Review of Vaccine Acceptance Rates. Vaccines.

[8] Campbell F., Archer B., Laurenson-Schafer H., Jinnai Y., Konings F., Batra N., Pavlin B., Vandemaele K., Van Kerkhove M.D., Jombart T. (2021). Increased transmissibility and global spread of SARS-CoV-2 variants of concern as at June 2021. Eurosurveillance.

[9] Neuzil K.M. (2021). Interplay between Emerging SARS-CoV-2 Variants and Pandemic Control. N. Engl. J. Med..

[10] Römmele C., Ebigbo A., Kahn M., Zellmer S., Muzalyova A., Hammel G., Breitling L.P., Bartenschlager C., Beyer A., Rosendahl J. (2021). SARS-CoV-2-Infektionsrisiko und Seroprävalenz bei Mitarbeitern des Gesundheitswesens in aerosolerzeugenden Tätigkeiten in Deutschland. Z. Gastroenterol..

[11] Römmele C., Ebigbo A., Kahn M., Zellmer S., Muzalyova A., Hammel G., Bartenschlager C., Beyer A., Rosendahl J., Schlittenbauer T. (2021). Health-care workers in gastrointestinal endoscopy are at higher risk for SARS-CoV-2 infection compared to other aerosol-generating disciplines. medRxiv.

[12] Mayer M., Zellmer S., Zenk J., Arens C., Ebigbo A., Muzalyova A., Thoelken R., Jering M., Kahn M., Breitling L.P. (2021). Status quo after one year of COVID-19 pandemic in otolaryngological hospital-based departments and private practices in Germany. Eur. Arch. Otorhinolaryngol..

[13] Zellmer S., Kahn M., Ebigbo A., Muzalyova A., Classen J., Grünherz V., Böser J., Breitling L.P., Beyer A., Rosendahl J. (2021). Ein Jahr Covid-19: Testung, Verwendung von Schutzausrüstung und Auswirkungen auf die Gastrointestinale Endoskopie in Deutschland. Z. Gastroenterol..

[14] (2021). Robert-Koch-Institut: SurvStat@RKI 2.0. https://survstat.rki.de/.

[15] Bundesamt D.S. (2021). Bevölkerungsstand: Amtliche Einwohnerzahl Deutschlands 2021. https://www.destatis.de/DE/Themen/Gesellschaft-Umwelt/Bevoelkerung/Bevoelkerungsstand/_inhalt.html.

[16] Lazarus J.V., Ratzan S.C., Palayew A., Gostin L.O., Larson H.J., Rabin K., Kimball S., El-Mohandes A. (2021). A global survey of potential acceptance of a COVID-19 vaccine. Nat. Med..

[17] Neumann-Bohme S., Varghese N.E., Sabat I., Barros P.P., Brouwer W., van Exel J., Schreyögg J., Stargardt T. (2020). Once we have it, will we use it? A European survey on willingness to be vaccinated against COVID-19. Eur. J. Health Econ..

[18] Dror A.A., Eisenbach N., Taiber S., Morozov N.G., Mizrachi M., Zigron A., Srouji S., Sela E. (2020). Vaccine hesitancy: The next challenge in the fight against COVID-19. Eur. J. Epidemiol..

[19] Gagneux-Brunon A., Detoc M., Bruel S., Tardy B., Rozaire O., Frappe P., Botelho-Nevers E. (2021). Intention to get vaccinations against COVID-19 in French healthcare workers during the first pandemic wave: A cross-sectional survey. J. Hosp. Infect..

[20] Biswas N., Mustapha T., Khubchandani J., Price J.H. (2021). The Nature and Extent of COVID-19 Vaccination Hesitancy in Healthcare Workers. J. Community Health.

[21] Martin C., Montesinos I., Dauby N., Gilles C., Dahma H., Van Den Wijngaert S., De Wit S., Delforge M., Clumeck N., Vandenberg O. (2020). Dynamics of SARS-CoV-2 RT-PCR positivity and seroprevalence among high-risk healthcare workers and hospital staff. J. Hosp. Infect..

[22] Kambhampati A.K., O’Halloran A.C., Whitaker M., Magill S.S., Chea N., Chai S.J., Kirley P.D., Herlihy R.K., Kawasaki B., Meek J. (2020). COVID-19-Associated Hospitalizations Among Health Care Personnel—COVID-NET, 13 States, March 1–May 31, 2020. MMWR Morb. Mortal. Wkly. Rep..

[23] Delbaere K., Close J.C., Brodaty H., Sachdev P., Lord S.R. (2010). Determinants of disparities between perceived and physiological risk of falling among elderly people: Cohort study. BMJ.

[24] Gao Z., Xu Y., Sun C., Wang X., Guo Y., Qiu S., Ma K. (2021). A systematic review of asymptomatic infections with COVID-19. J. Microbiol. Immunol. Infect..

[25] WHO (2020). WHO Concept for Fair Access and Equitable Allocation of COVID-19 Health Products. https://www.who.int/docs/default-source/coronaviruse/who-covid19-vaccine-allocation-final-working-version-9sept.pdf.

[26] Shehab M., Alrashed F., Shuaibi S., Alajmi D., Barkun A. (2021). Gastroenterological and hepatic manifestations of patients with COVID-19, prevalence, mortality by country, and intensive care admission rate: Systematic review and meta-analysis. BMJ Open Gastroenterol..

[27] da Rosa Mesquita R., Francelino Silva Junior L.C., Santos Santana F.M., de Oliveira T.F., Campos Alcantara R., Monteiro Arnozo G., da Silva Filho E.R., dos Santos A.G.G., da Cunha E.J.O., de Aquino S.H.S. (2021). Clinical manifestations of COVID-19 in the general population: Systematic review. Wien. Klin. Wochenschr..

