# Supplementary material for: SARS-CoV-2 Vaccination Rate and SARS-CoV-2 Infection of Health Care Workers in Aerosol-Generating Medical Disciplines
Source: J Clin Med. 2022 May 12;11(10):2751. doi: 10.3390/jcm11102751 (PMC9144158; doi:10.3390/jcm11102751)
Supplement: Supplementary file 1 [file jcm-11-02751-s001.zip › jcm-1702105-supplementary.pdf]

Supplementary materials
S1. Questionnaire: CoREM-NUM survey
University Hospital Augsburg

1. Basic data

1.1 Have you already participated in the 1st part of survey?
- Yes
- No

1.2 In which federal state is your medical facility located?
- Baden-Wuerttemberg
- Bavaria
- Berlin
- Brandenburg
- Bremen
- Hamburg
- Hesse
- Mecklenburg-Western Pomerania
- Lower Saxony
- North Rhine-Westphalia
- Rhineland-Palatinate
- Saarland
- Saxony
- Saxony-Anhalt
- Schleswig-Holstein
- Thuringia

1.3 What are the first two digits of your facility's zip code?

_ _

1.4 Please enter below the first two letters of your mother's maiden name and the first two letters of your father's first name.

(This code is used to better allocate the two-sided questionnaire to each other and does not allow any conclusions to be drawn about your person/practice/department)

_ _ _ _

1.5 Type of facility
- Hospital
- Private practice *(medical facility without bed occupancy)*

1.6 Which specialty do you belong to?
- Gastrointestinal Endoscopy
- Otorhinolaryngology
- Dental Medicine
- Maxillofacial Surgery
- Other: ___________

1.7 How many procedures did you perform on average per year before the start of the pandemic?
- <1000
- 1001-2000
- 2000-3000
- 3001-4000
- 4001-5000
- 5001-6000

- 6001-7000
- >7001

1.8 How many employees work in your respective department (e.g. endoscopy department/ entire private practice)?

1.9 How many employees were infected with SARS-CoV-2 in Q1 and Q2 2021?

- ___________________________
- None

2. Protective Measures

2.1 What prophylactic protective measures have been added to the pre-pandemic personal protective equipment?

- Mouth-nose protection
- FFP2/N95 or FFP3 masks
- Water repellent gowns
- Goggles/visor
- Ventilation between procedures
- Other: _____________________

2.2 Have you changed prophylactic personal protection measures of your employees compared to 2020?

- Yes
- No

2.3 What are the reasons for the change in protective measures?

- Variants of concern
- Availability of protective equipment
- Vaccination of the workforce
- Other: ____________________________________

2.4 How often you and your employees used/use the following personal protective measures

2.4.1 In the 1. calendar quarter 2021 (01.-03.2021):

a) Mouth-nose-protection	b) FFP2/N95 or FFP3 masks	c) Protective gown	d) Protective goggles or /Visor?	e) Ventilation between procedures
- always	- always	- always	- always	- always
- mostly	- mostly	- mostly	- mostly	- mostly
- often	- often	- often	- often	- often
- rarely	- rarely	- rarely	- rarely	- rarely
- never	- never	- never	- never	- never

2.4.2 In the current calendar quarter (since 04.2021):

a) Mouth-nose-protection	b) FFP2/N95 or FFP3 masks	c) Protective gown	d) Protective goggles or /Visor?	e) Ventilation between procedures
- always	- always	- always	- always	- always
- mostly	- mostly	- mostly	- mostly	- mostly
- often	- often	- often	- often	- often
- rarely	- rarely	- rarely	- rarely	- rarely
- never	- never	- never	- never	- never

2.4 By how much have procedures become more time-consuming due to your hygiene concept/additional prophylactic protective measures?

- By < 25%
- By >25% bis 50%
- By > 50 -75%
- By 100%
- Procedures have not become more time-consuming

3. Pre-interventional testing

3.1 Has the strategy of pre-interventional testing of patients for SARS-CoV-2 changed compared to 2020?

- No
- Yes

3.1.2 Which reasons are responsible for the change?

- Variants of consents
- Corona Test Offensive
- Availability of tests
- Other: ______________________________

3.2 What method did you use to test your patients pre-interventionally or what test did you ask for?

a) Year 2020 (Q2 –Q4)	b) Year 2021 (Q1-Q2)
- We tested/test pre-interventionally for SARS-CoV-2 predominantly by PCR	- We tested/test pre-interventionally for SARS-CoV-2 predominantly by PCR.
- We tested/test pre-interventionally for SARS-CoV-2 predominantly by antigen test	- We tested/test pre-interventionally for SARS-CoV-2 predominantly by antigen test.
- We requested/request mainly an externally performed current PCR test for SARS-CoV-2	- We requested/required mainly an externally performed current PCR test for SARS-CoV-2
- We requested/required mainly an externally performed current antigen test for SARS-CoV-2	- We requested/required mainly an externally performed current antigen test for SARS-CoV-2
- We did/do not perform pre-interventional testing for SARS-CoV-2	- We did/do not perform pre-interventional testing for SARS-CoV-2

4. HCW testing

4.1 Do you offer your employees the opportunity to be tested for SARS-CoV2 infection?

- Yes
- No

4.2 For which reasons?

- Variants of concerns
- Corona Test Offensive
- Availability of tests
- Other: ______________________________

4.3 Which method can your employees use to get tested for SARS-CoV-2?

- PCR
- Antigen rapid test
- Antibody test
- Other: ____________________________________

4.4 How often can your employees get tested for SARS-CoV-2?
- Daily
- Weekly
- Testing is the responsibility of the employee
- Other: ____________________________________

5. Infections among HCW

5.1 How many employees were already infected by SARS-CoV-2?

____

5.2 How many of the SARS-CoV-2 infected employees were most likely infected in the following situations?
- In private environment
- At work (e.g. during interventions)
- At work (other patient contact, e.g., registration)
- At work (without patient contact, e.g., during break)
- Unclear source of infection

5.3 Did fully vaccinated employees get infected with SARS-CoV-2?
- Yes
- No
- Unknown

5.4 How many employees get infected with SARS-CoV-2 in following time frame?
- < 1 week after becoming fully-vaccination
- > 1 week after becoming fully-vaccination

6. Financial implications of the COVID-19 pandemic

6.1 Are your increased expenses due to your hygiene concept sufficiently covered by billing flat rates or other funds? *(Question only for private practices)*
- Yes
- No

6.2 To what extent did you suspend procedures due to the pandemic?

a) In the 1. calendar quarter 2021 (01.-03.2021)

- Not at all
- Cancellation of up to 50% of elective procedures
- Cancellation of more than 50% of elective procedures
- Cancellation of all elective procedures

b) In the current calendar quarter

- Not at all
- Cancellation of up to 50% of elective procedures
- Cancellation of more than 50% of elective procedures
- Cancellation of all elective procedures

6.3 What were/are the predominant reasons for the cancellation of elective procedures?

a) In the 1. calendar quarter 2021 (01.-03.2021)

- Voluntarily preventive
- Ordered by the health department
- Due to staff shortage
- Due to patient shortage

b) In the current calendar quarter

- Voluntarily preventive
- Ordered by the health department
- Due to staff shortage
- Duo to patient shortage

7. Vaccination progress among HCW

7.1 How high do you asses the percentage of fully-vaccinated employees in your medical department/private practice?

a) Physicians
- below 20 %
- 20 % to < 40 %
- 40 % to < 60 %
- 60 % to < 80 %
- 80 % to 100 %

b) Nurses
- below < 20 %
- 20 % to < 40 %
- 40 % to < 60 %
- 60 % to < 80 %
- 80 % to 100 %

7.2 Does the percentage of fully-vaccinated employees in your medical department/private practice meet your expectations?

a) Physicians
- Yes, completely
- No, it exceeds my expectations
- No, it is below my expectations

b) Nurses
- Yes, completely
- No, it exceeds my expectations
- No, it is below my expectations

7.3 Are you vaccinated against SARS-CoV-2?
- Yes
- No
- No answer

7.4 Do you feel to be at higher risk of SARS-CoV-2 infection due to your occupation compared to the general population?
- Totally agree
- Rather true
- Neutral
- Rather disagree
- Totally disagree

7.5 Did you feel to be at higher risk of infection due to your professional activity compared to the general population prior to your vaccination?
- Totally agree
- Rather true
- Neutral
- Rather disagree
- Totally disagree

7.6 Have your concerns regarding increased risk of SARS-CoV-2 infection been reduced by vaccination?
- Yes
- No

7.7 Which aspects of your professional activity do you consider to be particular risk factors?
- Close contact with patients
- Facial /aerosol-generating procedures
- Long stay in the same room with patients
- Contact frequency due to professional activity
- Frequent asymptomatic courses of SARS-CoV-2 infection
- Uncompliant patients
- Low sensitivity of rapid antigen tests
- Mutations of the virus

8. Support in the defeating the COVID-19 pandemic

8.1 Was it possible to supply protective equipment in an adequate manner (price/quantity) for you and your workforce at the beginning of the pandemic?

- Totally agree
- Rather true
- Neutral
- Rather disagree
- Totally disagree

8.2 Did you have sufficient information material at the beginning of the pandemic to create a hygiene concept for your medical department/private practice?

- Totally agree
- Rather true
- Neutral
- Rather disagree
- Totally disagree

8.3 Did you have sufficient support creating a hygiene concept for your medical department/private practice?

- Totally agree
- Rather true
- Neutral
- Rather disagree
- Totally disagree

9. Feedback

In your opinion, what would be important to manage effectively in the event of a reoccurrence of a pandemic?

____
____
____
____
____
____
____

Supplementary materials S2: Vaccination rate depending on perceived risk of SARS-CoV-2 infection

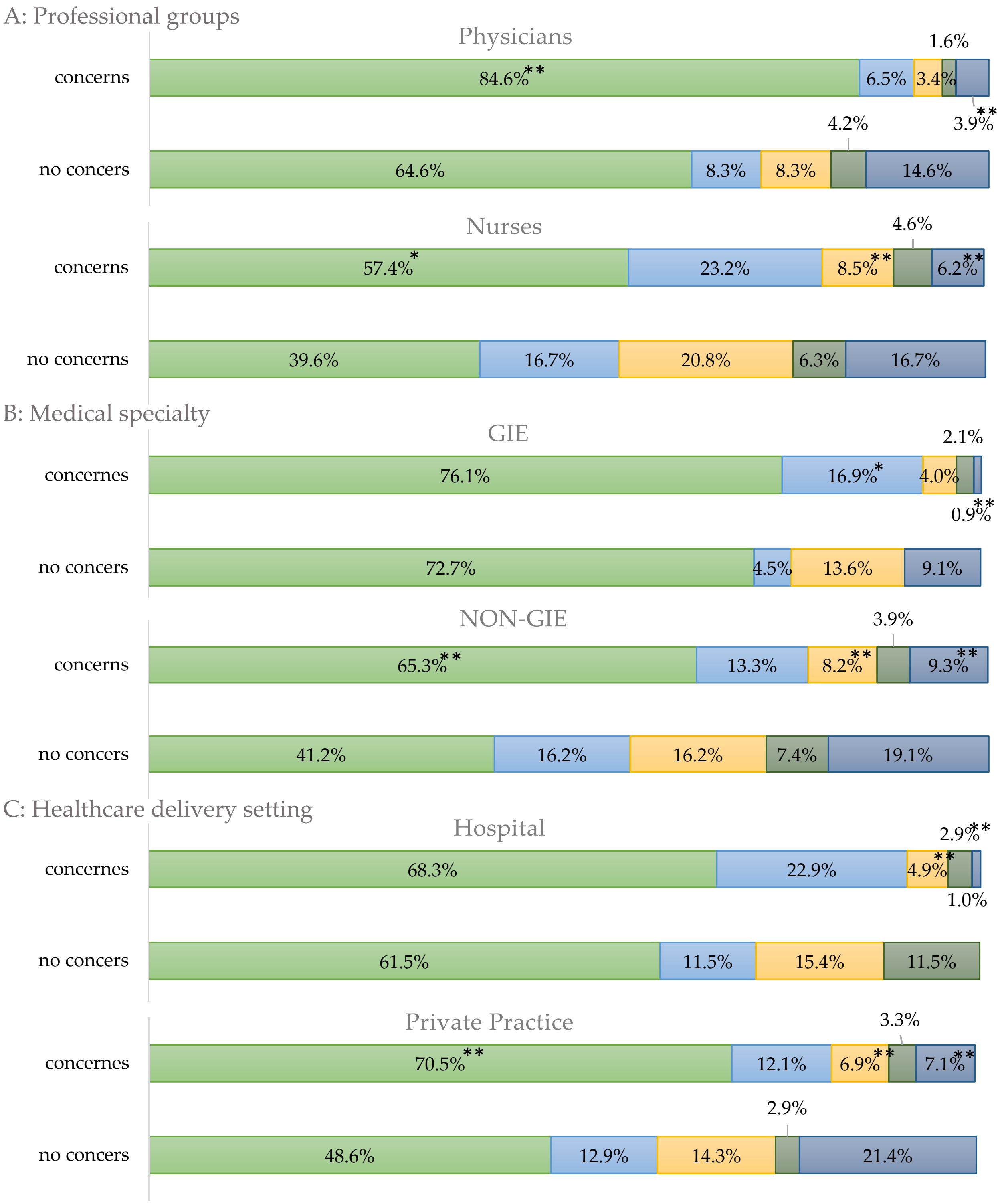

Figure S1: Vaccination rate depending on perceived risk of SARS-CoV-2 infection compared with the general population: (a) vaccination rate by professional group; (b) Vaccination rate by medical specialty; (c) Vaccination rate by health care delivery setting.
GIE: gastrointestinal endoscopy; other aggregated aerosol-generating specialties such as otolaryngology, oral- and maxillofacial surgery and dental medicine.
** Significance level p <0.01; * Significance level p <0.05
